# Transcranial temporal interference stimulation of the thalamus in a patient with disorders of consciousness: a case report

**DOI:** 10.3389/fnhum.2026.1788356

**Published:** 2026-04-29

**Authors:** Jiaojiao Peng, Leiyao Zhang, Wensheng Huang, Linghao Chen, Qingyu Liu, Yazhuo Kong, Pu Wang

**Affiliations:** 1 Department of Rahabilitation Medicine, The Seventh Affiliated Hospital of Sun Yat-Sen University, Shenzhen, Guangdong, China; 2 New Laboratory of Pattern Recognition, Institute of Automation, Chinese Academy of Sciences, Beijing, China; 3 State Key Laboratory of Cognitive Science and Mental Health, Institute of Psychology, Chinese Academy of Sciences, Beijing, China; 4 Department of Psychology, University of Chinese Academy of Sciences, Beijing, China; 5 Department of Radiology, The Seventh Affiliated Hospital of Sun Yat-sen University, Shenzhen, Guangdong, China

**Keywords:** deep brain stimulation, neuromodulation, prolonged disorders of consciousness, temporal interference stimulation, thalamus, vegetative state/unresponsive wakefulness syndrome

## Abstract

**Background:**

Prolonged disorders of consciousness (pDoC) are conditions with impaired consciousness lasting over 28 days, including coma, vegetative state/unresponsive wakefulness syndrome (VS/UWS), and minimally conscious state (MCS). Existing treatments offer limited benefit. Although thalamic deep brain stimulation (DBS) has shown potential to improve cognitive and behavioral function, its invasiveness limits clinical use. Transcranial temporal interference stimulation (TIS)—a noninvasive method using dual slightly different frequency currents to target deep brain regions, offers a potential alternative. We therefore conducted a clinical trial to explore the feasibility and potential effects of bilateral thalamic TIS in a patient with pDoC.

**Case presentation:**

A 40-year-old male patient experienced sudden cardiac arrest (CA) while playing basketball. Cranial magnetic resonance imaging (MRI) revealed abnormal signal changes in the bilateral caudate nuclei, putamen, and thalamus, consistent with ischemic–hypoxic brain injury secondary to CA. At the time of study enrollment, the patient had a Coma Recovery Scale-Revised (CRS-R) score of 4, indicating an early stage of the VS/UWS. Bilateral transcranial TIS targeting the thalamus was administered for 8 weeks, during which behavioral, neurophysiological, and imaging outcomes were systematically assessed. Improvements in CRS-R scores, alterations in EEG spectral power, and structural changes were observed over the intervention period.

**Conclusion:**

This study provides preliminary evidence supporting the feasibility of thalamus-targeted TIS in a patient with pDoC, representing an initial step toward clinical translation and further investigation of TIS in disorders of consciousness (DoC) neurorehabilitation. Our findings suggest that thalamic TIS may be associated with improved consciousness in this VS/UWS patient and highlights the need for future hypothesis-testing studies to establish its therapeutic potential.

## 1 Introduction

Prolonged disorders of consciousness (pDoC) refer to a category of disorders of consciousness (DoC) in which impaired consciousness persists for more than 28 days (Kondziella et al., 2020). The causes of pDoC are more relevant to traumatic brain injury, stroke, and cardiac arrest with attempted cardiac pulmonary resuscitation. Within the spectrum of DoC, distinct conditions include coma, vegetative state/unresponsive wakefulness syndrome (VS/UWS), and minimally conscious state (MCS) (Naccache, 2018). Coma represents a profound absence of arousal and awareness, lacking any response to external stimuli. VS/UWS involves intermittent wakefulness without self-awareness or environmental recognition, often accompanied by eye-opening movements (Owen and Coleman, 2008; Schnakers et al., 2009). MCS patients exhibit consistent clinical movement, but their state of awareness is unstable, recently classified into MCS − (displaying low-level behavioral responses) and MCS + (demonstrating high-level language-dependent responses) (Bender et al., 2015; Bruno et al., 2011; Edlow et al., 2021). Patients with pDoC may be bedridden for a long time causing several and multiple complications such as hydrocephalus, pulmonary infection, urinary tract infection, and/or paroxysmal sympathetic hyperactivity. These complications not only pose major challenges for caregivers but also impose significant economic burdens on families and society.

Positive outcomes remain scarce despite treatment efforts, including audiovisual and tactile stimulation, pharmacological treatment, noninvasive electromagnetic stimulation, and hyperbaric oxygen therapy (Thibaut et al., 2019). The challenge of identifying effective interventions for patients with impaired consciousness persists, highlighting the necessity for advancing research and therapeutic strategies in this crucial field. Recent advancements in neuromodulation techniques hold promise for enhancing consciousness and cognitive function. Deep brain stimulation (DBS), an invasive neurosurgical neuromodulation technique, has demonstrated promising results in patients with DoC (Schiff et al., 2007). A notable advantage of neuromodulation is its ability to precisely target specific brain regions and networks, thereby markedly enhancing network activity and accelerating functional recovery in patients with DoC (Afrasiabi et al., 2021; Edlow et al., 2021). The thalamus, a crucial hub in the brain connectome, plays a critical role in sustaining consciousness and wakefulness. DBS targeting the thalamus has been demonstrated to improve cognitive and behavioral functions in patients with DoC (Arnts et al., 2024; Arnts et al., 2022). However, the invasive nature and variable clinical outcomes of most surgical interventions for consciousness restoration have hindered their translation into standard clinical practice and limit their practical applicability (Deli and Green, 2024).

Overall, it is significant and necessary to develop novel neuromodulation techniques with both high spatial resolution and noninvasive character (Liu et al., 2021). Grossman et al. (2017) proposed a novel noninvasive brain stimulation technique—“temporally interfering” (TI) electrical stimulation. Rather than targeting a brain region with implanted electrodes, transcranial temporal interference stimulation (TIS) employs dual electric currents at slightly different frequencies, utilizing the interference pattern produced within the brain to create a low-frequency envelope capable of selectively stimulating deep brain regions. In recent years, TIS has emerged as a promising noninvasive alternative to DBS, offering notable advantages, particularly the avoidance of surgical implantation. Accumulating evidence supports the positive effects of TIS on neural function. For instance, TIS targeting the hippocampus has been shown to enhance memory performance in healthy participants (Violante et al., 2023). Moreover, striatal TIS significantly improves motor learning in older adults, with its efficacy depending on stimulation frequency—80 Hz TIS may counteract motor learning enhancement (Vassiliadis et al., 2024). Importantly, clinical trials indicate that TIS is a promising noninvasive therapeutic approach for Parkinson’s disease (PD) (Yang et al., 2025) and epilepsy (Missey et al., 2026). Together with prior animal research (Vieira et al., 2024), these human studies provide convergent evidence that TIS can safely and effectively modulate neuronal activity in deep brain structures.

Based on the above evidence, we hypothesized that bilateral thalamic TIS may produce therapeutic effects comparable to those of DBS in patients with pDoC. Accordingly, we conducted this study to evaluate the safety and potential effects of TIS for pDoC. In addition, we discuss multimodal approaches for assessing patients with pDoC, including clinical behavioral scales, electrophysiological assessments, and neuroimaging techniques.

## 2 Case presensation

**Figure 1:**
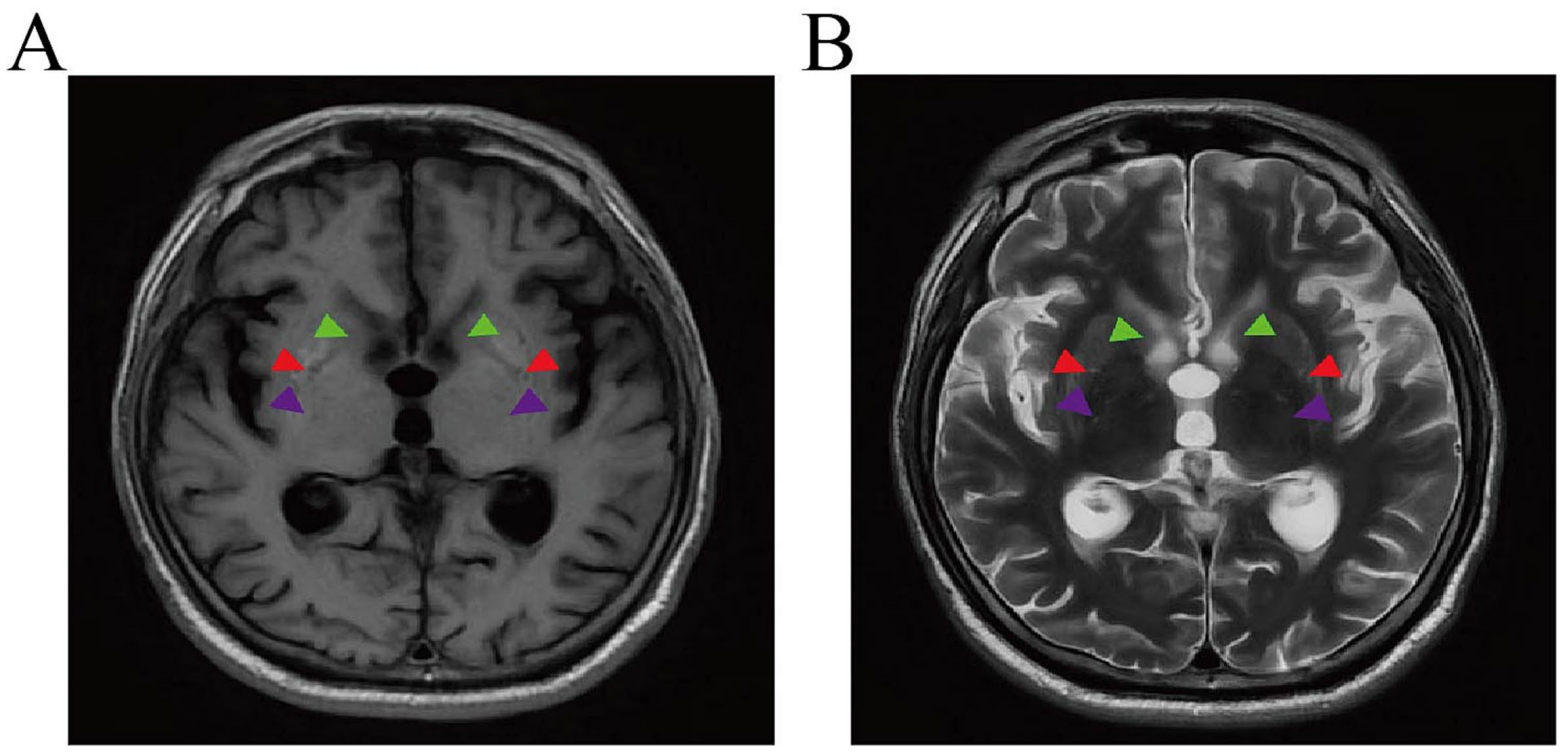
Abnormal signals in the bilateral caudate nucleus, putamen, and thalamus on T1-weighted **(A)** and T2-weighted **(B)** sequences. Green arrow: caudate nucleus; red arrow: putamen; purple arrow: thalamus.

A 40-year-old male patient suffered sudden cardiac arrest (CA) while playing basketball. Cranial MRI showed abnormal signals in the bilateral caudate nucleus, putamen, and thalamus (Figures 1A,B), consistent with ischemic–hypoxic brain injury secondary to CA. The TIS intervention began on day 39 after CA (week 0). Prior to enrollment, the patient was treated at an external hospital (days 0–15) and subsequently at our institution (days 16–30). Medical records and family reports indicated no clear improvement in consciousness during this period. Around day 20 post-event, the patient was diagnosed with VS/UWS, with a Coma Recovery Scale-Revised (CRS-R) score of 4. He exhibited spontaneous eye opening and limb withdrawal in response to pain but showed no behavioral evidence of awareness, consistent with the baseline status at treatment initiation. Conventional treatments, including pharmacotherapy, hyperbaric oxygen therapy, acupuncture, and exercise therapy, were maintained without modification throughout the study period.

**Figure 2:**
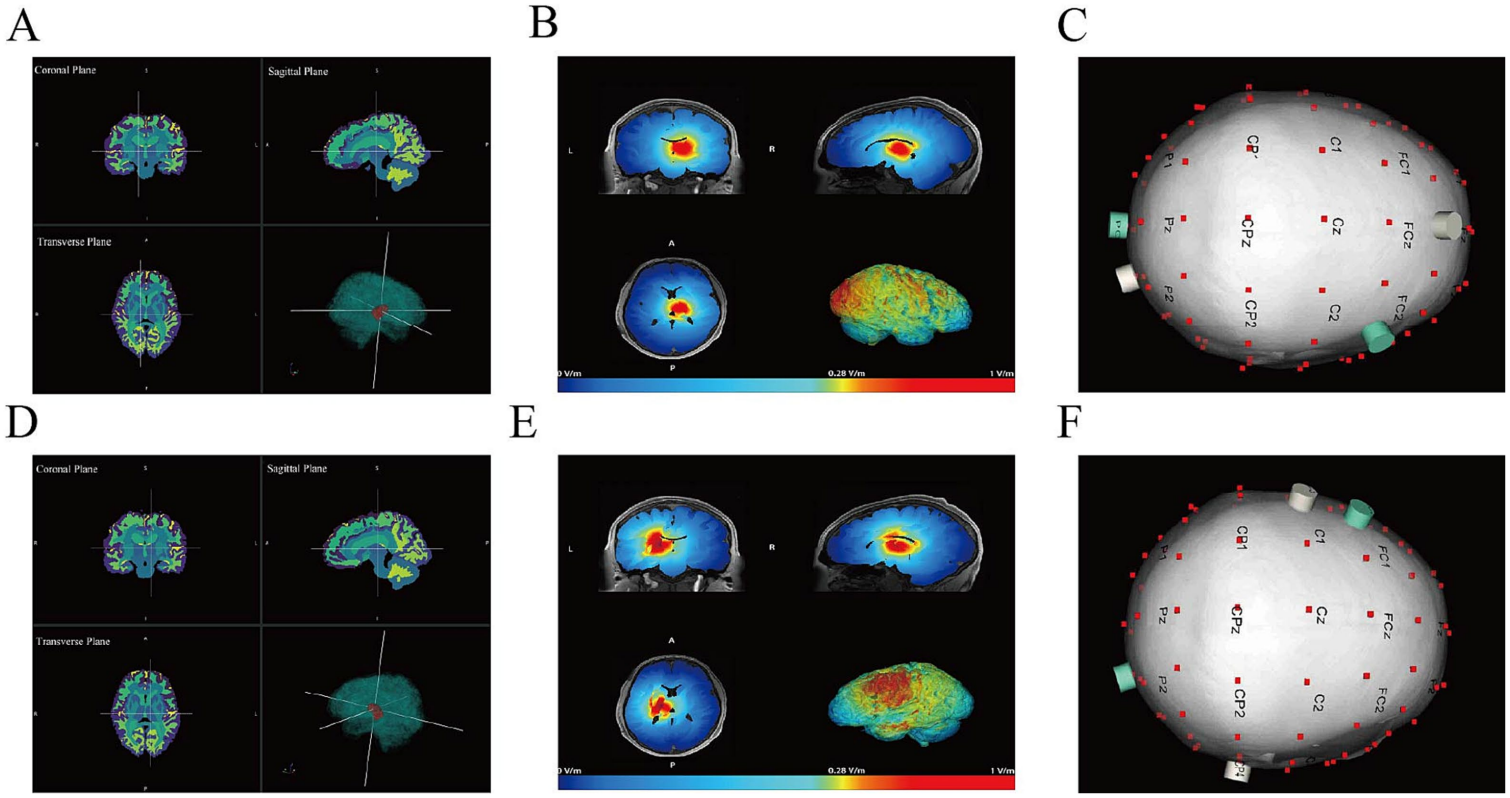
Thalamic TIS optimization using MOVEA. **(A,D)** Right **(A)** and left **(D)** thalamus selected as target regions. **(B,E)** Electric field distributions in the right **(B)** and left **(E)** thalamus derived from the MOVEA-optimized TIS protocol. **(C,F)** Electrode pair configuration scheme for the right **(C)** and left **(F)** thalamus.

The TIS device was developed by NeuraPlus. In this study, we applied the MOVEA algorithm to optimize the electrode montage and corresponding current intensities for TIS (Wang et al., 2023). According to the simulation results (Figure 2), right thalamus stimulation used electrode pairs O2-Fz/Oz-FC4 (Figure 2C), and left thalamus used TP8-C3/O2-FC3 (Figure 2F). The first and second electrode pairs delivered two independent stimulation waves with a frequency difference of 100 Hz and a base frequency of 2000 Hz, with fixed current intensity (5 mA peak-to-baseline) and target electric field strength (0.9 V/m) (Figures 2B,E). Electrodes were 0.848 cm^2^. Stimulation parameters were selected based on previous human TIS studies (Demchenko et al., 2025), which commonly employ carrier frequencies around 2 kHz and envelope frequencies within the beta (approximately 20 Hz) or gamma (30–130 Hz) bands, with current amplitudes of 1–3 mA per channel and stimulation durations of 10–30 min. Accordingly, a 2 kHz carrier frequency and a 100 Hz envelope frequency were applied, given the association of gamma-band activity with thalamocortical network function and consciousness. Stimulation was delivered once daily, five days per week. Each stimulation block lasted continuously for 20 min, including a 30-s ramp-up period at the beginning and a 30-s ramp-down period at the end. Each session consisted of 20 min of right thalamic stimulation followed by 20 min of left thalamic stimulation. The 8-week intervention was conducted under continuous medical supervision.

The Coma Recovery Scale-Revised (CRS-R), a gold-standard tool for assessing DoC, ranges from 0 to 23 points, with higher scores reflecting greater levels of consciousness. CRS-R assessments were performed by trained and experienced assessors blinded to the study procedures. During the initial evaluation, his CRS-R score was 4. After 4 weeks of TIS treatment, the CRS-R score improved markedly to 9, representing a 5-point increase from baseline. The patient demonstrated sound localization, such as orienting toward family members’ voices, smiling in response to auditory stimuli, and exhibiting emotional reactions while watching videos on a mobile device. By the end of the 8-week treatment period, the CRS-R score further increased to 12. The patient not only produced nonverbal vocalizations spontaneously but also displayed attentive behaviors during family conversations, including sustained eye contact and head turning toward sound sources, indicative of improved auditory-attention network connectivity. Detailed CRS-R itemized scores are provided in Supplementary Table S1. Supplementary videos demonstrating the patient’s behavioral responses are provided for peer-review purposes only, in accordance with patient privacy and ethical guidelines.

**Figure 3:**
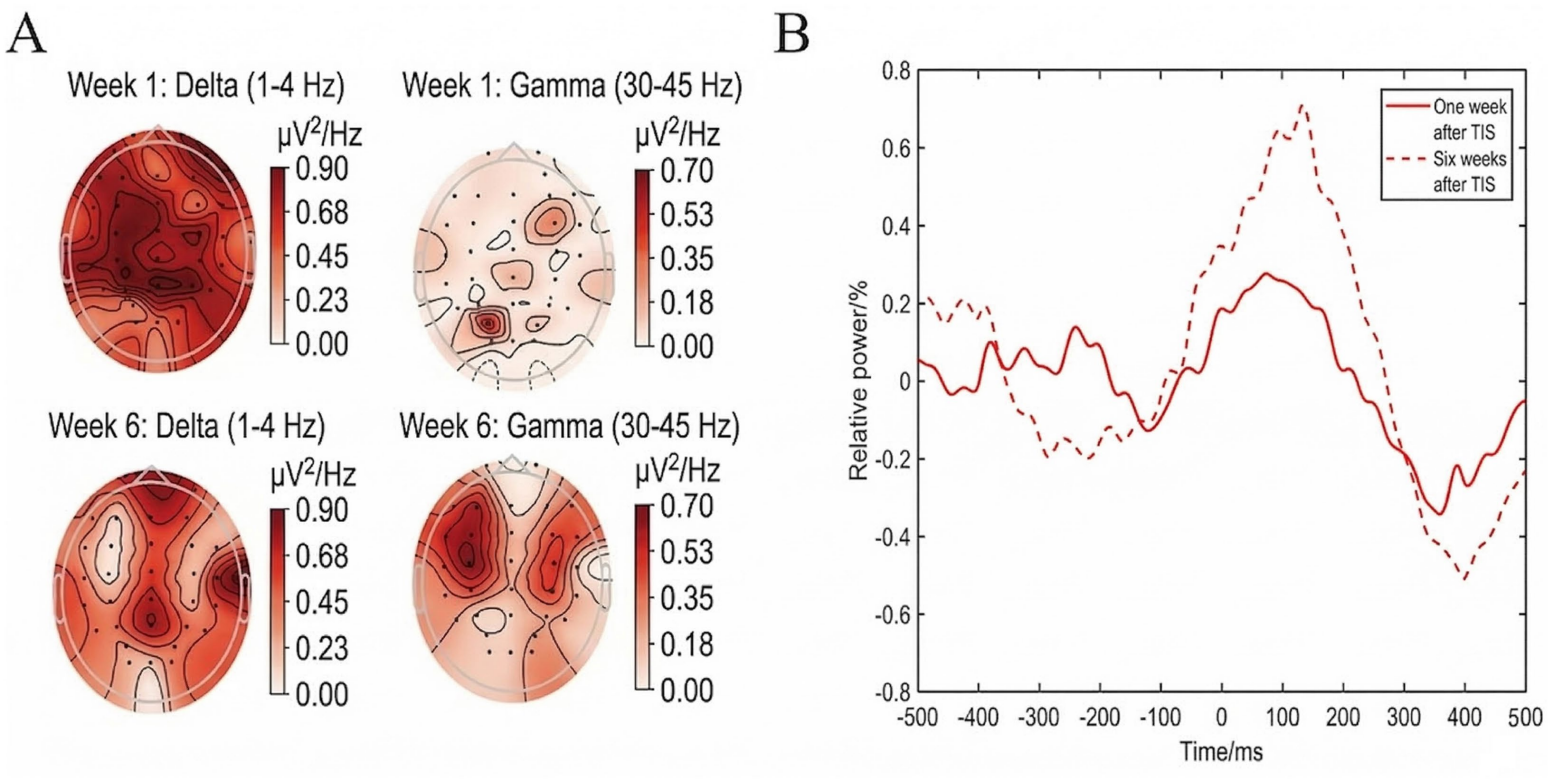
**(A)** Whole-brain relative spectral maps. With ongoing TI treatment, delta power decreased and gamma power markedly increased. **(B)** Power changes at 1 and 6 weeks after TIS treatment relative to baseline. With treatment progression, alpha-band power increased.

A 32-channel electroencephalography (EEG) system (NeuraPlus) was employed to evaluate TIS efficacy. EEG data analysis methods are in Supplementary material. For the spectral analysis, we focused on the delta band (1–4 HZ) and the gamma band (30–45 HZ). We found that after the TIS treatment, the relative powers of the patient with pDoC showed a decreasing trend in delta-band and an increasing trend in gamma-band (Figure 3A). Moreover, this trend became more pronounced as the treatment duration increased. Using admission data as baseline, we compared the patient’s data after 1 week and 6 weeks of TIS treatment. As shown in Figure 3B, with increasing treatment duration, the patient exhibited power change rate (PCR) in the alpha band, indicating a potential gradual recovery of consciousness. Although the total clinical observation period was initially designated as 8 weeks, the primary intensive TIS intervention and systematic EEG monitoring were specifically centered on the first 6 weeks of the recovery phase. Preliminary data analysis indicated that the neurophysiological shifts reached their peak at week 6 during hospitalization. Therefore, week 6 was selected as the primary endpoint to evaluate the maximal cumulative efficacy of the core intervention. Extending the primary comparative analysis to week 8 could potentially introduce confounding variables associated with the later discharge phase and changes in routine care, which might dilute the specific effects of the intensive TIS.

**Figure 4:**
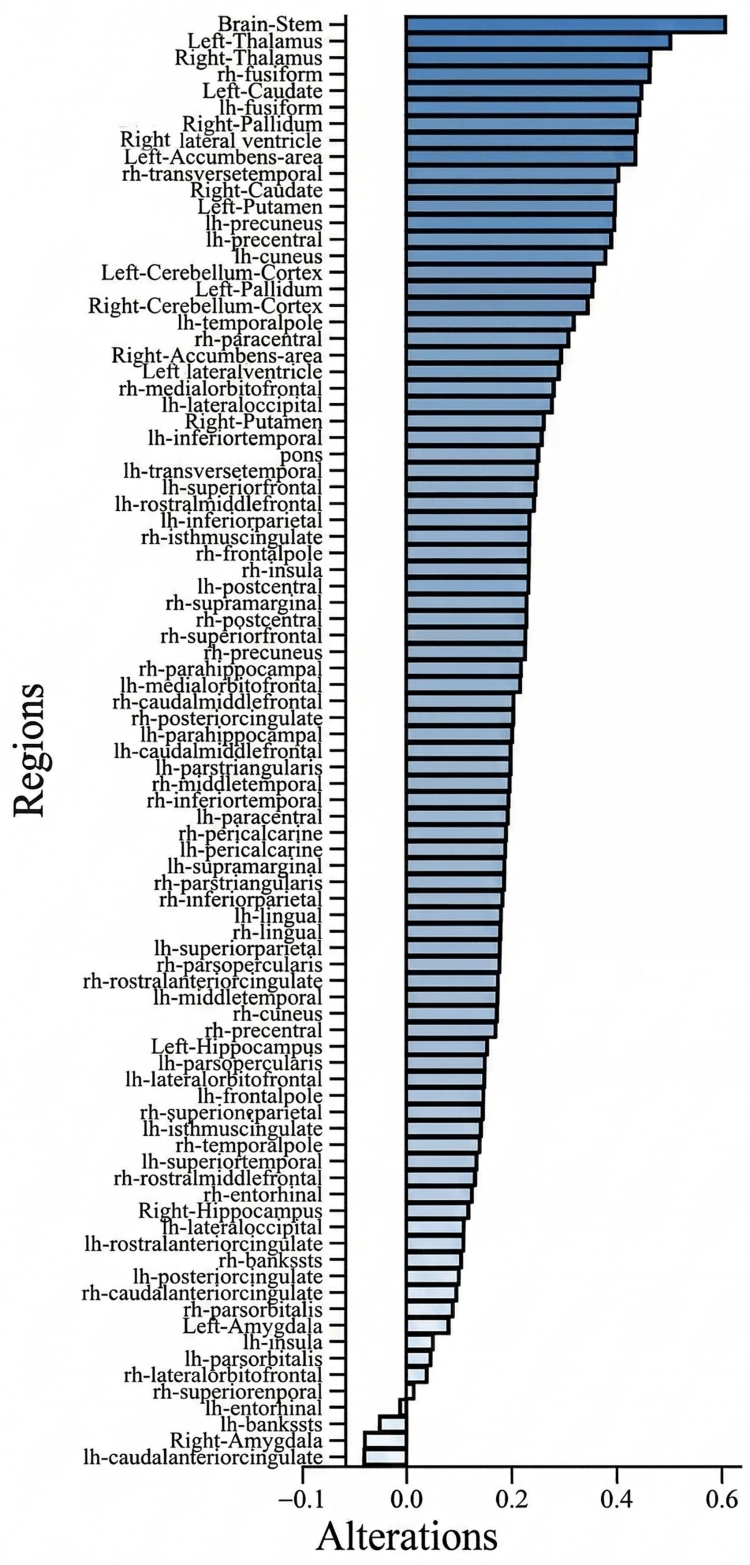
Structural remodeling across subcortical and cortical regions following 8 weeks of TIS. The bar chart illustrates the descriptive alterations represented as percentage change in regional brain volumes compared to the baseline (admission). Regions are grouped by functional networks. Note: As this is a single-case study, data represent observational morphometric shifts for this individual patient, and no statistical hypothesis testing was performed. The most pronounced volumetric increases, such as those in the brainstem and thalamus, are discussed in the context of the stimulation targets.

MRI offers high spatial resolution and superior integration with structural information. The assessment of structural brain changes, including cortical thickness and subcortical volumetric analysis, has been increasingly validated as a sensitive proxy for neuroplasticity and pathological alterations. Recent large-scale and clinical studies have demonstrated the robustness of these morphometric markers in characterizing brain maturation and structural deviations in neuropsychiatric cohorts (Wu et al., 2025; Yu et al., 2023). Building on this methodological framework, our study employs these validated tools to track the longitudinal structural remodeling during the 8-week TIS treatment. MRI data analysis methods are in Supplementary material. Regarding volumetric changes in subcortical and cortical nuclei, as TIS treatment progressed and the patient showed signs of recovery, most subcortical and cortical regions exhibited an overall increasing trend. Among these, the most pronounced volumetric increases were observed in the brainstem, thalamus, caudate nucleus, cerebellar cortex, and right pallidum (Figure 4).

## 3 Discussion

This study aimed to explore the feasibility and potential effects of thalamus-targeted TIS in a patient with pDoC (VS/UWS) by assessing behavioral, neurophysiological, and imaging outcomes. Over the 8-week intervention period, the patient’s CRS-R score increased from 4 to 12. This change, particularly in the CRS-R arousal subscore, may reflect improvements in cognitively mediated behaviors related to executive function.

Our preliminary observations suggest that notable resting-state EEG changes may be associated with TIS administration: relative delta-band (1–4 Hz) power decreased significantly, whereas gamma-band (30–45 Hz) power increased markedly—consistent with other noninvasive neuromodulation studies. For example, Tarantino et al. reported that nodal tDCS administered over the left dorsolateral prefrontal cortex (DLPFC) reduced low-frequency (delta/theta) activity and enhanced higher-frequency (alpha/beta) activity in patients with DoC, suggesting a reversal of pathological EEG slowing (Tarantino et al., 2024). The dose–response relationship observed in our data—whereby TIS facilitated recovery of consciousness—is consistent with progressive neuromodulatory effects reported in a tACS study demonstrating cumulative gamma-band enhancement with extended stimulation durations (Lee and Tramontano, 2025). Moreover, He et al. found that responders to rTMS exhibited a significant reduction in delta-band power, reinforcing the notion that attenuation of slow oscillations is a reliable electrophysiological marker of consciousness recovery (He et al., 2020).

The observed post-treatment enlargement of subcortical and cortical nuclei likely reflects activity-dependent neuroplasticity in coma recovery (Warren et al., 2025). As core ascending arousal system components, brainstem and thalamus volumetric expansion may indicate restored connectivity and neuromodulatory drive—critical for wakefulness recovery (Wang et al., 2025). The caudate nucleus, a key node within the fronto-striatal circuitry that supports executive functions and goal-directed behavior, exhibited volumetric gains, potentially underlying improved cognitive responsiveness. The cerebellar cortex, traditionally linked to motor coordination, also involved in attention and language, showed volumetric increases linked to severe brain injury functional recovery. Enlargement of the right pallidum—a key component of the cortico-striatum-pallidus circuit, playing a core role in motor control, cognitive function and emotional regulation—may reflect reinstated motivational drive and affective processing, aiding rehabilitation engagement. Meanwhile, the observed volumetric increases in subcortical nuclei may represent the activation of latent compensatory pathways, a phenomenon increasingly recognized in various forms of brain injury. For instance, recent research on subcortical stroke has highlighted the importance of dynamic inter-hemispheric interactions and structural adaptations in functional restitution (Fan et al., 2026). Similarly, the use of large-scale morphometric markers has elucidated how structural variations can reflect the brain’s resilience or risk profiles in compromised states (Kuang et al., 2023). Our preliminary observations suggest that TIS, by targeting deep-seated structures such as the thalamus, may induce similar structural remodeling, potentially reversing atrophic trends and re-establishing functional connectivity in patients with DoC. Collectively, these structural adaptations, observed in this single case may represent preliminary imaging correlates of favorable neurological changes, highlighting the potential value of targeting subcortical arousal and cortico-striatal-cerebellar circuits in coma-related interventions.

However, the interpretation of longitudinal volumetric increases observed in this single case should be approached with caution. Although these structural alterations temporally align with the patient’s clinical and electrophysiological improvements, alternative non-neuroplastic explanations cannot be excluded. For example, physiological fluid shifts, such as cerebrospinal fluid (CSF) redistribution or late-stage resolution of micro-edema, may subtly alter tissue boundaries. In addition, methodological factors should be considered; automated MRI analysis pipelines are susceptible to segmentation variability, and slight differences in head positioning, scanner noise, or patient hydration status between sessions may contribute to the observed volumetric variations. Future studies incorporating larger cohorts and multimodal imaging validation are needed to distinguish true stimulation-induced structural remodeling from these physiological and methodological confounds.

Reported adverse events associated with TIS across studies include mild sensations such as tingling and itching (Violante et al., 2023; Wang et al., 2024; Yang et al., 2024). In this case report, no complications or detectable adverse effects related to TIS were observed during the 8-week follow-up period. Our findings support the safety and feasibility of TIS in a patient with severe brain injury, representing an initial step toward clinical translation and further investigation of TIS in neurorehabilitation for patients with pDoC. However, several limitations of the current study should be acknowledged. First, the generalizability of these findings remains limited due to the single-subject design of this study. In addition, spontaneous recovery or the effects of ongoing rehabilitation cannot be excluded in this case report. Previous studies have reported that spontaneous recovery from the VS beyond one month after injury occurs in approximately 30% of patients by 6 months and 43% by 12 months post-injury (Vanhoecke and Hariz, 2017). Giacino and Kalmar reported that 51% of patients with VS demonstrated spontaneous recovery by 6 months, with only a modest increase observed at 12 months (Giacino and Kalmar, 1997). The prognosis for the minimally conscious state (MCS) is substantially more favorable than that for VS. For example, Lammi et al. reported that 83% of patients (15/18) emerged from MCS by 6 months (Lammi et al., 2005). The interval between injury and TIS intervention is a crucial consideration, as it helps distinguish spontaneous recovery from TIS-related effects. Therefore, larger sample sizes and randomized controlled trials are required to validate the therapeutic potential of TIS and advance its clinical application. Future studies should enroll patients only after an adequate post-injury period—at least 6 to 12 months—to allow for spontaneous emergence from coma.

Second, our protocol was primarily designed to explore the cumulative, long-term neuroplastic changes associated with repeated TIS interventions over several weeks. Due to clinical workflow constraints and the need to minimize patient fatigue during the intensive rehabilitation period, routine resting EEG recordings were not performed immediately before and after each individual stimulation session. Consequently, the present study cannot definitively distinguish acute, transient effects immediately following stimulation from delayed network consolidation occurring between sessions. Future studies incorporating immediate pre- and post-session functional assessments are warranted to further elucidate the temporal dynamics and acute mechanisms of TIS.

Another limitation of the present study is the absence of an MRI scan immediately prior to the initiation of TIS (week 0). Although structural comparisons were performed between admission and 8 weeks post-treatment, some changes may have begun during the pre-stimulation period. However, given the high spatial correspondence between the TIS focal zone and regions showing significant volumetric increases (e.g., the thalamus), it is plausible that the observed structural remodeling was driven, at least in part, by the neuromodulatory effects of TIS.

## 4 Conclusion

To our knowledge, this is the first case report describing preliminary behavioral and neurophysiological changes associated with thalamus-targeted TIS in a patient with pDoC. Our observations suggest a potential association between TIS intervention and improved levels of consciousness in this patient diagnosed with VS/UWS. The noted increases in high-frequency EEG gamma-band (30–45 Hz) power and decreases in low-frequency delta-band (1–4 Hz) power provide hypothesis-generating evidence of the neuromodulatory effects of TIS. Additionally, the post-intervention increases in cortical and subcortical tissue volumes may reflect potential neural plasticity and structural remodeling, potentially related to the observed clinical changes. Given the single-case design and the ongoing natural recovery phase following hypoxic–ischemic injury, these findings should be interpreted with caution. Future research, ideally including randomized controlled trials, is warranted to further investigate the potential role of TIS as an experimental intervention for DoC.

## Data Availability

The raw data supporting the conclusions of this article will be made available by the authors, without undue reservation.
