# Supplementary material for: Transcranial temporal interference stimulation of the thalamus in a patient with disorders of consciousness: a case report
Source: Front Hum Neurosci. 2026 Apr 29;20:1788356. doi: 10.3389/fnhum.2026.1788356 (PMC13167994; doi:10.3389/fnhum.2026.1788356)
Supplement: Supplementary file 1 [file Data_Sheet_1.pdf]

CARE case report guidelines

CARE Checklist of information to include when writing a case report

(cc) BY-NC-ND

Topic	Item	Checklist item description	Reported on Line
Title	1	The diagnosis or intervention of primary focus followed by the words "case report"	lines54-62
Key Words	2	2 to 5 key words that identify diagnoses or interventions in this case report, including "case report"	lines 102-104
Abstract (no references)	3a	Introduction: What is unique about this case and what does it add to the scientific literature?	lines 77-83
	3b	Main symptoms and/or important clinical findings	lines 92-93
	3c	The main diagnoses, therapeutic interventions, and outcomes	lines 89-93
	3d	Conclusion—What is the main "take-away" lesson(s) from this case?	lines 94-99
Introduction	4	One or two paragraphs summarizing why this case is unique (**may include references**)	lines 131-176
Patient Information	5a	De-identified patient specific information.	lines 189-191
	5b	Primary concerns and symptoms of the patient.	lines 199-201
	5c	Medical, family, and psycho-social history including relevant genetic information	Not applicable
	5d	Relevant past interventions with outcomes	Not available
Clinical Findings	6	Describe significant physical examination (PE) and important clinical findings.	lines 273-337
Timeline	7	Historical and current information from this episode of care organized as a timeline	lines 192-201 and 270-272
Diagnostic Assessment	8a	Diagnostic testing (such as PE, laboratory testing, imaging, surveys).	lines 199-201
	8b	Diagnostic challenges (such as access to testing, financial, or cultural)	Not applicable
	8c	Diagnosis (including other diagnoses considered)	Not applicable
	8d	Prognosis (such as staging in oncology) where applicable	Not applicable
Therapeutic Intervention	9a	Types of therapeutic intervention (such as pharmacologic, surgical, preventive, self-care)	lines 202-204 and 270-272
	9b	Administration of therapeutic intervention (such as dosage, strength, duration)	lines 210-214 and 270-272
	9c	Changes in therapeutic intervention (with rationale)	Not applicable
Follow-up and Outcomes	10a	Clinician and patient-assessed outcomes (if available)	lines 277-377
	10b	Important follow-up diagnostic and other test results	Not available
	10c	Intervention adherence and tolerability (How was this assessed?)	line 271-272
	10d	Adverse and unanticipated events	line 549-551
Discussion	11a	A scientific discussion of the strengths AND limitations associated with this case report	lines 555-596
	11b	Discussion of the relevant medical literature **with references**.	lines 404-436 and 507-575
	11c	The scientific rationale for any conclusions (including assessment of possible causes)	lines 602-618
	11d	The primary "take-away" lessons of this case report (without references) in a one paragraph conclusion	lines 613-618
Patient Perspective	12	The patient should share their perspective in one to two paragraphs on the treatment(s) they received	Not applicable
Informed Consent	13	Did the patient give informed consent? Please provide if requested	Yes √ No ☐
