# Supplementary material for: Transcranial temporal interference stimulation of the thalamus in a patient with disorders of consciousness: a case report
Source: Front Hum Neurosci. 2026 Apr 29;20:1788356. doi: 10.3389/fnhum.2026.1788356 (PMC13167994; doi:10.3389/fnhum.2026.1788356)
Supplement: Supplementary file 2 [file Data_Sheet_2.docx]

*Supplementary Material*

## 1 Supplemental Methods

### 1.1 EEG

We employed a 32-channel electroencephalography (EEG) system (NeuraPlus) with a sampling rate of 250 Hz, following the 10-10 electrode placement system. C1 was designated as the reference electrode. Prior to each recording session, researchers, under the supervision of a physician, ensured that the impedance of all channels was maintained below 10 kΩ. Resting-state EEG signals were continuously acquired over a 5-minute duration following each weekly treatment session. Resting-state EEG data were acquired offline at baseline and approximately 5-minute duration after the TIS sessions to avoid any potential stimulation-induced electrical artifacts. Preprocessing included the use of independent component analysis (ICA) to remove ocular and myogenic artifacts, ensuring that the spectral analysis, particularly in the high-frequency gamma band (30–45 Hz), reflected genuine neural oscillations.

### 1.2 MRI

High-resolution T1-weighted (T1w) images were acquired on GE Architect with a 48-channel head coil, using a gradient echo sequence with the following parameters: TR = 2218 ms, TE = 2.5 ms, inversion time = 900 ms, flip angle = 10°, field of view=24 mm ×24 mm, slices = 176, slice thickness=1 mm, and voxel size=1 ×1 ×1 mm^3^.

## 2 Data Analysis

### 2.1 EEG

#### Data Preprocessing

EEG data were processed and analyzed mainly using Python 3.11 with the MNE 1.7.1 package. During preprocessing, power line interference at 50 Hz was removed using SciPy 1.11.4. A band-pass filter (1–100 Hz) was applied to attenuate low-frequency drift and high-frequency motion artifacts. Bad channels, ocular artifacts, and motion-related noise were identified and removed through manual inspection and ICA. The EEG data were then segmented into 2-second epochs, and epochs exhibiting extreme peak frequencies or excessive amplitude variations were excluded to ensure data quality.

#### Spectral Analysis

Spectral analysis was conducted on the preprocessed EEG data using the ‘multitaper’ method implemented in the MNE package. This method offers superior spectral concentration and reduced variance by averaging multiple tapered estimates, making it particularly suitable for analyzing neural oscillations with high temporal and frequency resolution. Absolute and relative power values were computed for the following frequency bands: delta (1–4 Hz), theta (4–8 Hz), alpha (8–13 Hz), beta (13–30 Hz), and gamma (30–100 Hz).

#### power change rate (PCR) Analysis

To assess the neuromodulatory effects of TIS, we analyzed the longitudinal changes in spectral power. We compared resting-state EEG data acquired at admission as baseline with follow-up recordings at one week and six weeks. The relative power changes were calculated to identify treatment-induced synchronization or desynchronization in specific frequency bands.

## 2.2 MRI

#### Image processing

First, the structural (T1w) images were processed using FreeSurfer version 7.3.2 (https://surfer.nmr.mgh.harvard.edu/), applying an automatic robust processing pipeline. The preprocessing steps included skull stripping, brain tissue segmentation, and surface reconstruction. Cortical surfaces were then parcellated into 68 regions of interest (ROIs) according to the Desikan-Killiany atlas. Subcortical volumes were automatically segmented during this process. The total intracranial volume (TIV) was also calculated for the participant, defined as the sum of the volumes of gray matter (GM), white matter (WM), and cerebrospinal fluid (CSF), and was included as a covariate in subsequent analyses. To measure the changes in different brain regions before and after treatment, we used the post-treatment data, subtracting the pre-treatment results and dividing by the pre-treatment results as an indicator of treatment effectiveness.

#### Comparison of cortical and subcortical segmentation

We assessed the efficacy of TIS treatment by evaluating changes in cortical and subcortical nucleus volumes in both hemispheres. To facilitate this comparison, volumetric data obtained before the initiation of TIS served as the baseline. The corresponding cortical and subcortical volumes measured after eight weeks of treatment were then calculated. The therapeutic effects of TIS were quantified using the rate of volumetric change relative to baseline.

$$\text{Rate}\text{=}\frac{\text{TIS}_{\text{post}}\text{-}\text{TIS}_{\text{pre}}}{\text{TIS}_{\text{pre}}}$$
