# Supplementary material for: Transcranial temporal interference stimulation of the thalamus in a patient with disorders of consciousness: a case report
Source: Front Hum Neurosci. 2026 Apr 29;20:1788356. doi: 10.3389/fnhum.2026.1788356 (PMC13167994; doi:10.3389/fnhum.2026.1788356)
Supplement: Supplementary file 3 [file Table_1.docx]

Table S1. Coma Recovery Scale–Revised (CRS-R) total and subscale scores at weeks 0, 4, and 8 of transcranial temporal interference stimulation (TIS).

Supplementary videos documenting the patient’s behavioral responses during the intervention period are provided for peer-review purposes only. To ensure patient privacy and confidentiality, these videos are restricted to the editorial review process and will not be made publicly.

| CRS-R subscales | Week 0 | Week 4 | Week 8 |
| --- | --- | --- | --- |
| Auditory | 0 | 2 | 2 |
| Visual | 0 | 3 | 3 |
| Motor | 2 | 2 | 2 |
| Oromotor/verbal | 0 | 0 | 2 |
| Communication | 0 | 0 | 0 |
| Arousal | 2 | 2 | 3 |
| CRS-R total scores | 4 | 9 | 12 |
